# Correction: Sun, Y.-D.; Yokomi, R. Genotype Sequencing and Phylogenetic Analysis Revealed the Origins of Citrus Yellow Vein Clearing Virus California Isolates. *Viruses* 2024, *16*, 188

**DOI:** 10.3390/v16040553

**Published:** 2024-04-01

**Authors:** Yong-Duo Sun, Raymond Yokomi

**Affiliations:** United States Department of Agriculture, Agricultural Research Service, San Joaquin Valley Agricultural Sciences Center, Parlier, CA 93648, USA

## Missing Citation

In the original publication [1], reference “21. Abrahamian, P.; Tian, T.; Posis, K.; Guo, Y.; Yu, D.; Blomquist, C.L.; Wei, G.; Adducci, B.; Vidalakis, G.; Bodaghi, S.; et al. Genetic analysis of the emerging citrus yellow vein clearing virus reveals a divergent virus population in American isolates. *Plant Dis.* **2023**. https://doi.org/10.1094/PDIS-09-23-1963-RE.” was not cited. The citation has now been inserted in 2. Materials and Methods, *2.1. Sample Collection, RNA Extraction, and Real-Time Quantitative Polymerase Chain Reaction (RT-qPCR)*, Paragraph two and should read: 

A duplex RT-qPCR for the simultaneous detection of CYVCV and the citrus Nad5 gene as an internal quality control was employed. Specifically, the RT-qPCR reaction took place in a 10 µL reaction volume composed of 2 µL of RNA template, 5 µL of 2× reaction buffer, 0.4 µL each of CYVCV forward primer (5′-AAA TCC ATT AAC ACA GTG ACC TTC C-3′) and reverse primer (5′-AAC TCC TGA CAG TGC TCC AA-3′), 0.1 µM of a CYVCV-specific 6-FAM/BHQ-1 labeled TaqMan probe (5′d FAM-CGTCGTTGCCAAGACACGCCA-BHQ-1), 0.4 µL each of Nad5 forward primer (5′-GATGCTTCTTGGGGCTTCTTKTT-3′) and reverse primer (5′-ACATAAATCGAGGGCTATGCGGATC-3′), and 0.1 µM of a Nad5-specific VIC/QSY labeled TaqMan probe (5′d VIC-CAT AAG TAG CTT GGT CCA TCT TTA TTCCAT-QSY), along with 0.2 µL of iScript advanced reverse transcriptase and 0.9 µL of double-distilled water. This mixture was placed into a PCR plate, with cycling conditions encompassing reverse transcription at 50 °C for 5 min, initial denaturation at 94 °C for 2 min, followed by 40 cycles of denaturation at 94 °C for 10 s, and annealing/extension at 60 °C for 40 s [21]. RNA samples at a concentration of 10 ng/µL were tested in triplicate. 

With the above correction, the reference citation numbers 21–37 have been changed to 22–38, respectively. The authors state that the scientific conclusions are unaffected. This correction was approved by the Academic Editor. The original publication has also been updated.
