# Supplementary material for: Genotype Sequencing and Phylogenetic Analysis Revealed the Origins of Citrus Yellow Vein Clearing Virus California Isolates
Source: Viruses. 2024 Jan 26;16(2):188. doi: 10.3390/v16020188 (PMC10891506; doi:10.3390/v16020188)
Supplement: Supplementary file 1 [file viruses-16-00188-s001.zip › viruses-2831575-supplementary.pdf]

Event No.	Recombinant	Major parent	Minor parent	Detection methods						
				R	G	B	M	C	S	T
1	KX156742.1	KX156735.1	KX156749.1	+	+	-	+	+	+	+
2	MW429491.1	MW429487.1	Unknown	-	-	-	+	+	+	+
3	KX156735.1	KX156738.1	JX040635.1	+	+	+	-	-	-	+
4	KT696512.1	Unknown	KX156742.1	-	+	-	+	+	+	-

Supporting Table S1. The recombination analysis of *Citrus yellow vein clearing virus* isolates upon genome sequences. Only four event were detected in over four different methods implemented in RDP4. Different algorithms abbreviation: R: RDP; G: GENECONV; B: BOOTSCAN; M: MAXCHI; C: CHIMAERA; S: SISCAN; T: 3SEQ.

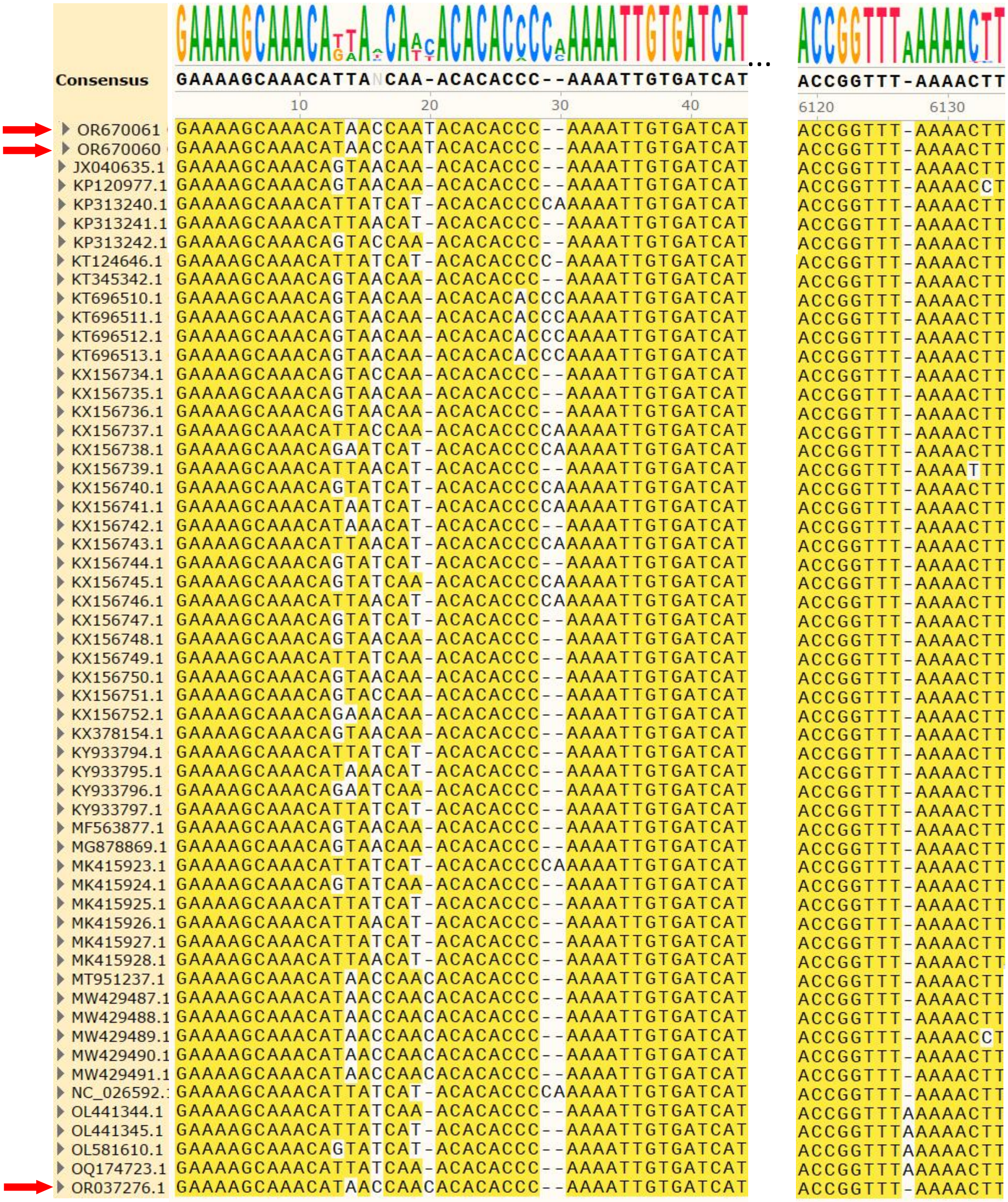

Supporting Figure S1. A snapshot of the insertion/deletion mutant sites via multiple sequence alignment of 57 CYVCV genome sequences. The CYVCV CA isolates were marked with red arrows.

A

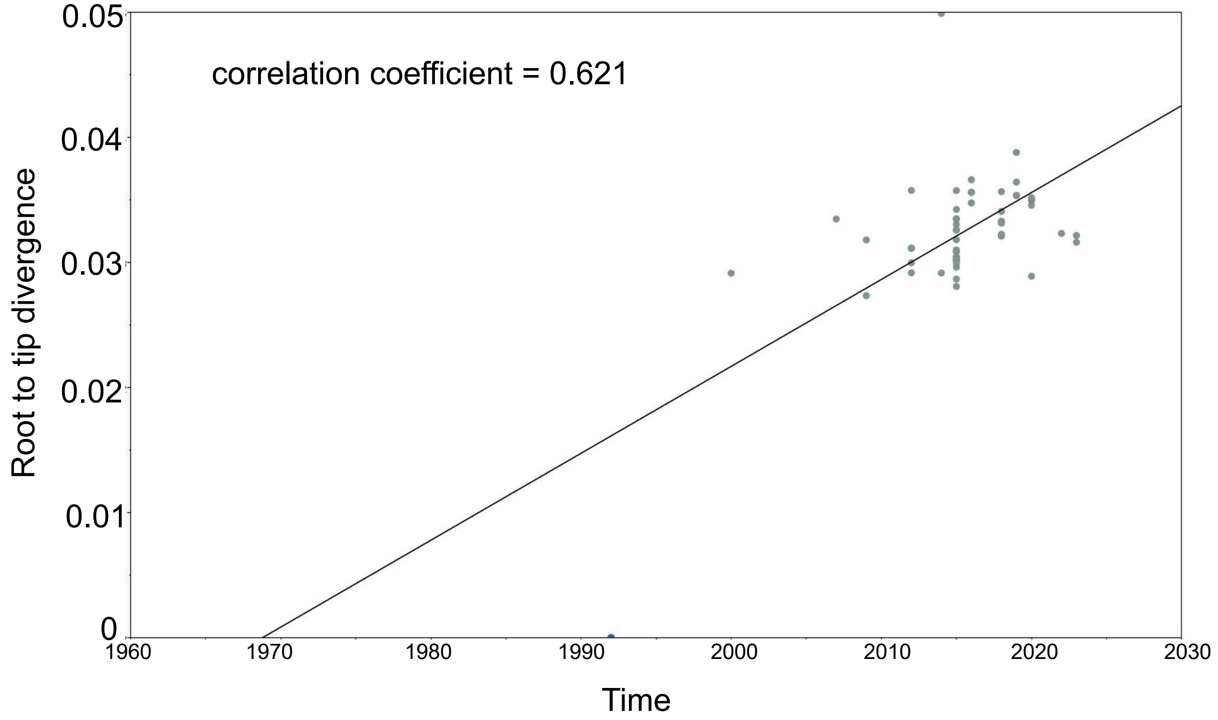

B

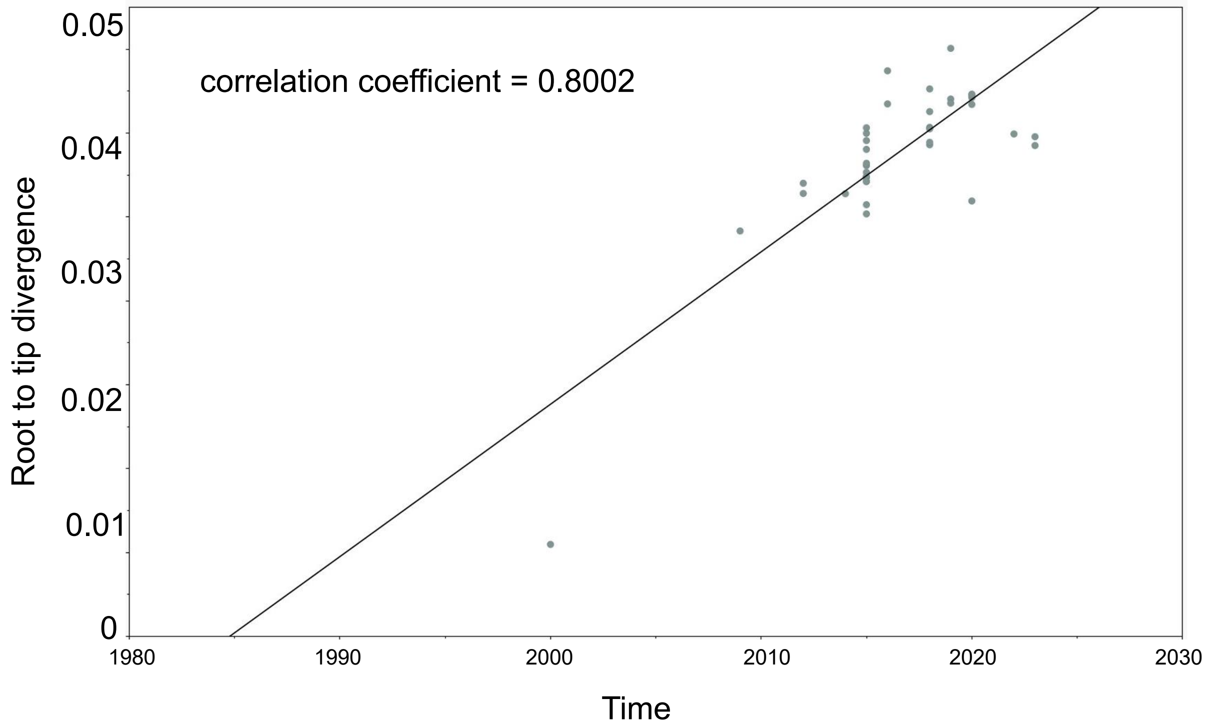

Supporting Figure S2. Root-to-tip divergence plot, as a function of sampling time for Maximum clade credibility tree clusters. **A.** Root-to-tip divergence plot of 53 nonrecombinant *Citrus yellow vein clearing virus* (CYVCV) genome sequences. Correlation coefficient equals to 0.621. **B.** 38 selected CYVCV genome sequences used for Bayesian phylodynamic inference in this study. The optimized Correlation coefficient equals to 0.8002.
